# Risk of Subsequent Primary Cancers Among Adult-Onset 5-Year Cancer Survivors in South Korea: Retrospective Cohort Study

**DOI:** 10.2196/48380

**Published:** 2024-05-08

**Authors:** Yoon Young Choi, Myeongjee Lee, Eun Hwa Kim, Jae Eun Lee, Inkyung Jung, Jae-Ho Cheong

**Affiliations:** 1 Department of Surgery Soonchunhyang Bucheon Hospital Soonchunhyang University College of Medicine Bucheon si Republic of Korea; 2 Department of Biomedical Systems Informatics Yonsei University College of Medicine Seoul Republic of Korea; 3 Department of Surgery Yonsei University Health System Yonsei University College of Medicine Seoul Republic of Korea

**Keywords:** cancer, survivors, subsequent primary cancer, adult, onset, primary cancer, risk, general population, screening, genetic testing, retrospective, cohort study, health Insurance, survivability, hereditary, FPC, SPC, subsequent cancer

## Abstract

**Background:**

The number of cancer survivors who develop subsequent primary cancers (SPCs) is expected to increase.

**Objective:**

We evaluated the overall and cancer type–specific risks of SPCs among adult-onset cancer survivors by first primary cancer (FPC) types considering sex and age.

**Methods:**

We conducted a retrospective cohort study using the Health Insurance Review and Assessment database of South Korea including 5-year cancer survivors diagnosed with an FPC in 2009 to 2010 and followed them until December 31, 2019. We measured the SPC incidence per 10,000 person-years and the standardized incidence ratio (SIR) compared with the incidence expected in the general population.

**Results:**

Among 266,241 survivors (mean age at FPC: 55.7 years; 149,352/266,241, 56.1% women), 7348 SPCs occurred during 1,003,008 person-years of follow-up (median 4.3 years), representing a 26% lower risk of developing SPCs (SIR 0.74, 95% CI 0.72-0.76). Overall, men with 14 of the 20 FPC types had a significantly lower risk of developing any SPCs; women with 7 of the 21 FPC types had a significantly lower risk of developing any SPCs. The risk of developing any SPC type differed by age; the risk was 28% higher in young (<40 years) cancer survivors (SIR 1.28, 95% CI 1.16-1.42; incidence: 30 per 10,000 person-years) and 27% lower in middle-aged and older (≥40 years) cancer survivors (SIR 0.73, 95% CI 0.71-0.74; incidence: 80 per 10,000 person-years) compared with the age-corresponding general population. The most common types of FPCs were mainly observed as SPCs in cancer survivors, with lung (21.6%) and prostate (15.2%) cancers in men and breast (18.9%) and lung (12.2%) cancers in women. The risks of brain cancer in colorectal cancer survivors, lung cancer in laryngeal cancer survivors, and both kidney cancer and leukemia in thyroid cancer survivors were significantly higher for both sexes. Other high-risk SPCs varied by FPC type and sex. Strong positive associations among smoking-related cancers, such as laryngeal, head and neck, lung, and esophageal cancers, were observed. Substantial variation existed in the associations between specific types of FPC and specific types of SPC risk, which may be linked to hereditary cancer syndrome: for women, the risks of ovarian cancer for breast cancer survivors and uterus cancers for colorectal cancer survivors, and for men, the risk of pancreas cancer for kidney cancer survivors.

**Conclusions:**

The varying risk for SPCs by age, sex, and FPC types in cancer survivors implies the necessity for tailored prevention and screening programs targeting cancer survivors. Lifestyle modifications, such as smoking cessation, are essential to reduce the risk of SPCs in cancer survivors. In addition, genetic testing, along with proactive cancer screening and prevention strategies, should be implemented for young cancer survivors because of their elevated risk of developing SPCs.

## Introduction

The number of cancer survivors is on the rise due to population growth, an aging population, and improved survival rates resulting from early diagnosis and advanced cancer treatment strategies [1]. As of 2019, there were approximately 16.9 million and 2 million cancer survivors in the United States and South Korea, respectively [2]. Managing the health of cancer survivors is critical because they face unique physical, psychosocial, and medical conditions, with the more serious being subsequent primary cancers (SPCs) [1,3]. Identifying the associations between the types of SPC and first primary cancer (FPC) is crucial, as cancer survivors continue to live in similar environments to where their FPC developed and the genetic risk persists. Identification of and preemptive intervention for these associations provide opportunities for risk reduction and early detection through tailored prevention and cancer screening programs.

A comprehensive study investigating SPCs among 5-year cancer survivors using the Surveillance, Epidemiology, and End Results (SEER) Program reported a higher risk of SPCs with several types of FPCs [4]. However, the SEER study had a predominantly White population (>80%), and over 40% of male and female participants were prostate and breast cancer survivors, respectively. In Korea, previous studies have mainly focused on specific types of FPCs, rather than comprehensively addressing cancer survivors in general [5-8]. Therefore, given the differences in ethnicity and cancer incidence between the United States and South Korea, a comprehensive epidemiological investigation is necessary to provide new insight on developing SPCs in cancer survivors with diverse socioenvironmental and different ethnic-genetic interactions.

The occurrence of cancers is influenced by environmental and hereditary factors, as well as random mutations [9]. Hereditary factors are linked to young-onset and multiple cancers [10-12]. Consequently, young cancer survivors may be at a higher risk of developing SPCs than their older counterparts and may exhibit unique combinations of FPCs and SPCs that are influenced by genetic susceptibility. Thus, age should be taken into consideration when investigating the association between FPCs and SPCs; however, this aspect has not been assessed in previous studies.

In this study, we assessed the relationships between FPCs and SPCs by analyzing data obtained from the South Korea Health Insurance Review and Assessment (HIRA) database, which covers nearly the entire Korean population.

## Methods

### Data Source and Study Population

The study was a nationwide, population-based, cohort study that analyzed data obtained from the HIRA database of South Korea between January 1, 2008, and December 31, 2019, which includes over 50 million Koreans. The study population included patients diagnosed with cancer between January 1, 2009, and December 31, 2010, and newly diagnosed cancer cases during this period were identified by excluding patients diagnosed with cancer in 2008. Cancers were classified into 23 types (20 types for men and 21 types for women) using the 10th revision of the *International Classification of Diseases* (*ICD*) code, and the Korean Individual Co-payment Beneficiaries Program (ICBP) data in the HIRA were used to define each cancer type. The ICBP was established for rare and intractable diseases, including cancers, in 2008, and patients in this program pay only 5% to 10% of their medical costs for 5 years from the day of registration. Therefore, the most affected individuals are registered in ICBP, and patients who were diagnosed with cancer before 2008 were retrospectively registered in the program. We followed those cancer patients until December 31, 2019. Cancer survivors were defined as cancer patients who lived longer than 5 years without developing other cancers with no evidence of recurrence of the first diagnosed cancers. SPCs were defined as a newly diagnosed, distinct cancer type, excluding same-type cancers to minimize the bias from misclassification of FPC recurrence. Survivor follow-up began 5 years after their FPC diagnosis and continued until death; loss to follow-up; December 31, 2019; or occurrence of an SPC, whichever came first. If a patient had multiple SPCs, the first type was considered the SPC. The primary outcomes were incidence (per 10,000 person-years) and relative risk (standardized incidence ratio [SIR]) of SPCs among 5-year survivors of FPCs. The Korean National Cancer Statistics 2015 [13], the middle year of the present cohort, was used to determine the expected cancer rates.

### Statistical Analysis

Incidence rates of SPCs among the overall sample and each group of survivors were calculated by dividing the observed number of SPCs by the corresponding total person-years and then multiplying by 10,000. To investigate the risk of SPCs among the overall sample and each group of survivors compared with the general Korean population, we calculated the age-standardized incidence ratio by type of FPCs and sex using the Korean National Cancer Statistics 2015 [13], the middle year of the cohort, as the expected cancer rates. The 95% CI of the SIR was calculated using the Poisson distribution. All estimates were calculated for overall SPCs and for type-specific SPCs. The 12 smoking-related cancers, 12 obesity-related cancers, 7 alcohol-related cancers, and 6 infection-related cancers were considered in the subgroup analyses to assess environmental factors for developing SPCs [14-16]. The incidences and SIRs were estimated to quantify the risk of developing any types of each environment factor–related SPC. A 2-sided *P*<.05 was considered statistically significant; multiple comparison corrections were not conducted, as the analyses were exploratory. SAS Enterprise Guide version 9.4 (SAS Institute Inc) was used for all statistical analyses.

### Ethical Considerations

This study was approved by the research ethics committee of the South Korea National Health Insurance Sharing Service (M20200902739) and the institutional review board of Severance Hospital (4-2020-0155). The need for informed consent was waived owing to the use of deidentified data. Access to the HIRA data is restricted to authorized individuals and is only permissible from designated computers.

## Results

**Figure 1:**
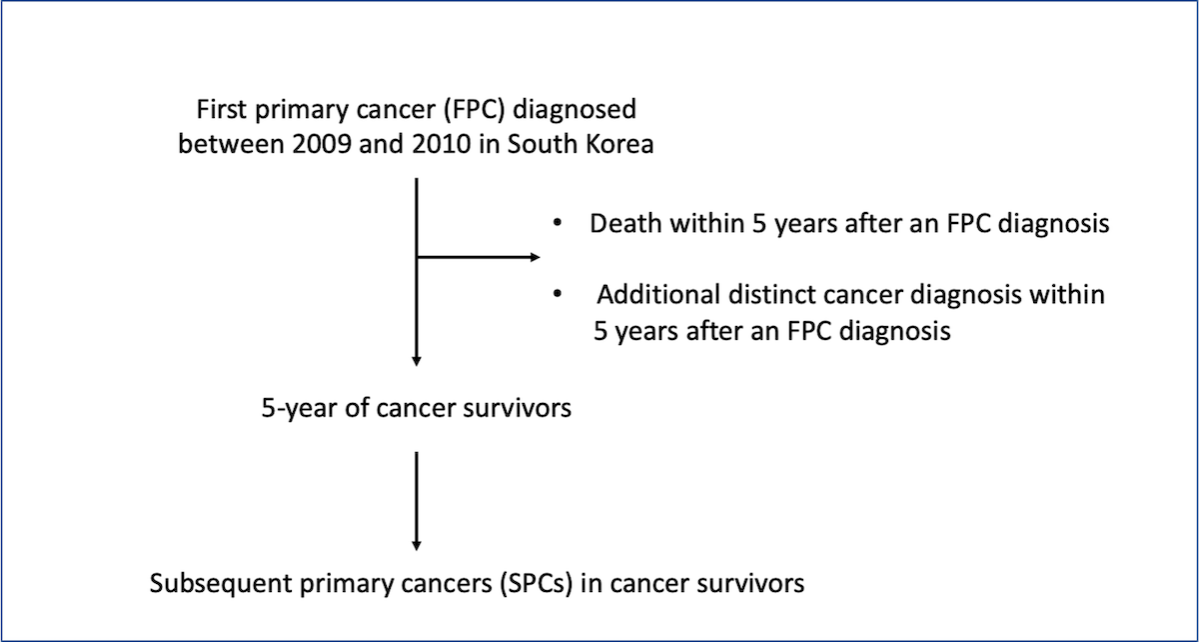
Flow diagram for patient selection.

A total of 266,241 (71.7%) 5-year cancer survivors were identified among 371,181 patients who were diagnosed with FPCs between 2009 and 2010, accruing 1,003,008 person-years of follow-up 5 years after their FPCs (mean 3.8 years; median 4.3 years; Figure 1 and Table 1). Among 371,181 patients who were diagnosed with an FPC between January 2009 and December 2010 in South Korea, there were 266,241 (71.7%) 5-year cancer survivors, and among the survivors, 7348 (2.8%) patients had SPCs. The percentage of 5-year survivors varied by the type of FPC; survival was lowest with pancreatic cancer (2628/7392, 35.6%) and highest with thyroid cancer (66,087/69,211, 95.5%; Table S1 in Multimedia Appendix 1). Of the survivors, 56.1% (149,352/266,241) were female, and 12.5% (33,171/266,241) and 30.1% (80,249/266,241) were younger than 40 years and older than 65 years, respectively.

Among all survivors, 7348 SPC cases (73 per 10,000 person-years) were identified, representing a 26% lower risk of developing SPCs (SIR 0.74, 95% CI 0.72-0.76; Table 2). There were 4207 and 3141 SPC cases (99 and 54 per 10,000 person-years) in male and female survivors, respectively, which corresponded to 34% and 11% lower risks of developing SPCs compared with the risk in the general Korean population. When considering age at FPC diagnosis, the risk of developing SPCs differed by age, with a higher risk observed among young patients with FPCs (<40 years old), and the risk gradually decreased with age (Table S2 in Multimedia Appendix 1). Therefore, subgroups by age were considered for further analysis. The risk of developing SPCs was significantly higher when the patient had FPC before they reached 40 years old (men: SIR 1.43, 95% CI 1.10-1.84; women: SIR 1.26, 95% CI 1.12-1.40), while the risk was significantly lower in patients with an FPC at ≥40 years old (men: SIR 0.66, 95% CI 0.64-0.68; women: SIR 0.86, 95% CI, 0.83-0.89).

Overall, many survivor groups defined by FPC types and sex had lower-than-expected risks of developing SPCs compared with the risks in the Korean general population. Specifically, men with 14 of the 20 FPC types had a significantly lower risk of developing any SPCs, while women with 7 of the 21 FPC types had a significantly lower risk of developing any SPCs (Table 3).

**Table 1:** Characteristics of survivors diagnosed with a first primary cancer in 2009-2010 at age ≥18 years among 5-year survivors in South Korea.

Characteristic		Survivors, n (%)	Person-years of follow-up at 5 years after the first primary cancer		
			Total	Mean	Median
Total sample		266,241 (100)	1,003,008	3.8	4.3
**Sex**					
	Men	116,889 (43.9)	424,836	3.6	4.2
	Women	149,352 (56.1)	578,172	3.9	4.3
**Age at first primary cancer diagnosis (years)**					
	18-39	33,171 (12.5)	128,648	3.9	4.3
	40-49	54,689 (20.5)	210,840	3.9	4.3
	50-64	98,132 (36.9)	367,554	3.7	4.3
	≥65	80,249 (30.1)	295,966	3.7	4.2
**Calendar year of first primary cancer diagnosis**					
	2009	135,195 (50.8)	574,434	4.2	4.9
	2010	131,046 (49.2)	428,574	3.3	3.8

**Table 2:** Risk of developing any type of subsequent primary cancer (SPC) among 5-year cancer survivors with their first diagnosis between 2009 and 2010 in South Korea.

Category		Survivors, n (%)	Observed SPCs, n (%)	Expected SPCs, n	Incidence per 10 000 person-years	SIR^a^ (95% CI)
**Overall**						
	Age at FPC^b^ <40 years	33,171 (12.5)	381 (5.2)	297	30	1.28 (1.16-1.42)
	Age at FPC ≥40 years	233,070 (87.5)	6,967 (94.8)	9614	80	0.73 (0.71-0.74)
	Total	266,241 (100)	7348 (100)	9912	73	0.74 (0.72-0.76)
**Men**						
	Men with age at FPC <40 years	8219 (7)	62 (1.5)	43	20	1.43 (1.10-1.84)
	Men with age at FPC ≥40 years	10,8670 (93)	4145 (98.5)	6326	106	0.66 (0.64-0.68)
	Total	116,889 (43.9)	4207 (57.3)	6369	99	0.66 (0.64-0.68)
**Women**						
	Women with age at FPC <40 years	24,952 (16.7)	319 (10.2)	254	33	1.26 (1.12-1.40)
	Women with age at FPC ≥40 years	124,400 (83.3)	2822 (89.8)	3289	59	0.86 (0.83-0.89)
	Total	149,352 (56.1)	3141 (42.7)	3542	54	0.89 (0.86-0.92)

^a^SIR: standardized incidence ratio.

^b^FPC: first primary cancer.

**Table 3:** Risk of developing any type of subsequent primary cancers (SPCs) among 5-year survivors of a first primary cancer (FPC), with their first diagnosis between 2009 and 2010 in South Korea, by the type of their FPC.

FPC		Survivors, n (%)	Age at FPC (years), mean	Age at SPC (years), mean	Observed SPCs, n (%)	Incidence per 10,000 person-years	Expected SPCs, n	SIR^a^ (95% CI)
**Men**								
	Head and neck	2315 (2)	56.4	68.4	95 (2.3)	108	126	0.75 (0.61-0.92)
	Esophagus	1458 (1.2)	64.9	73.3	63 (1.5)	111	120	0.52 (0.40-0.67)
	Stomach	30,165 (25.8)	60.4	71.9	1286 (30.6)	108	1750	0.73 (0.70-0.78)
	Colorectal	22,008 (18.8)	60.8	71.6	1071 (25.5)	126	1322	0.81 (0.76-0.86)
	Liver	10,116 (8.7)	57.8	67.7	129 (3.1)	42	438	0.30 (0.25-0.35)
	Gallbladder and biliary	1334 (1.1)	64.4	71.0	44 (1)	85	105	0.42 (0.30-0.56)
	Pancreas	1420 (1.2)	63.4	69.7	26 (0.6)	44	116	0.23 (0.15-0.33)
	Larynx	1438 (1.2)	62.9	72.3	132 (3.1)	248	103	1.28 (1.07-1.51)
	Lung	10,349 (8.9)	65.5	72.3	158 (3.8)	40	712	0.22 (0.19-0.26)
	Breast	111 (0.1)	56.5	73.4	8 (0.2)	192	6	1.34 (0.58-2.64)
	Prostate	10,628 (9.1)	68.0	76.2	362 (8.6)	132	566	0.64 (0.58-0.71)
	Testis	408 (0.3)	33.3	55.8	6 (0.1)	38	4	1.50 (0.55-3.27)
	Kidney	3482 (3.0)	55.4	67.7	156 (3.7)	127	163	0.96 (0.81-1.12)
	Bladder	5033 (4.3)	63.0	74.2	252 (6.0)	155	307	0.82 (0.72-0.93)
	Brain	1687 (1.4)	46.9	64.8	23 (0.5)	40	60	0.38 (0.24-0.58)
	Thyroid	10,515 (9)	46.5	64.2	300 (7.1)	75	287	1.05 (0.93-1.17)
	Hodgkin	164 (0.1)	43.1	74.7	3 (0.1)	47	5	0.60 (0.12-1.75)
	Non-Hodgkin	2358 (2)	52.9	66.8	76 (1.8)	87	110	0.69 (0.55-0.87)
	Multiple myeloma	491 (0.4)	61.5	64.3	3 (0.1)	27	23	0.13 (0.03-0.38)
	Leukemia	1409 (1.2)	47.5	62.0	14 (0.3)	34	46	0.31 (0.17-0.51)
	Total	116,889 (100)	59.6	71.2	4207 (100)	99	6369	0.66 (0.64-0.68)
**Women**								
	Head and neck	1176 (0.8)	54.5	63.1	25 (0.8)	57	33	0.76 (0.49-1.13)
	Esophagus	138 (0.1)	64.9	64.0	4 (0.1)	74	5	0.75 (0.21-1.93)
	Stomach	15,182 (10.2)	60.2	70.2	366 (11.7)	60	475	0.77 (0.69-0.86)
	Colorectal	14,610 (9.8)	61.9	69.7	394 (12.5)	68	448	0.88 (0.80-0.97)
	Liver	3062 (2.1)	61.9	69.5	41 (1.3)	43	84	0.49 (0.35-0.66)
	Gallbladder and biliary	1349 (0.9)	65.8	70.9	30 (1)	57	51	0.59 (0.40-0.85)
	Pancreas	1208 (0.8)	64.7	67.8	12 (0.4)	24	48	0.25 (0.13-0.44)
	Larynx	102 (0.1)	61.6	70.6	7 (0.2)	186	3	2.03 (0.81-4.17)
	Lung	4210 (2.8)	63.9	72.3	49 (1.6)	33	128	0.38 (0.28-0.51)
	Breast	29,866 (20)	50.6	62.6	599 (19.1)	53	608	0.99 (0.91-1.07)
	Cervix	8901 (6)	51.9	63.3	222 (7.1)	60	249	0.89 (0.78-1.02)
	Uterus	2529 (1.7)	51.7	61.5	80 (2.5)	79	68	1.18 (0.94-1.47)
	Ovary	3089 (2.1)	48.7	59.8	80 (2.5)	72	68	1.18 (0.93-1.46)
	Kidney	1739 (1.2)	57.7	69.8	54 (1.7)	84	52	1.03 (0.77-1.35)
	Bladder	1146 (0.8)	65.3	72.5	37 (1.2)	94	39	0.95 (0.67-1.31)
	Brain	1813 (1.2)	52.3	61.2	40 (1.3)	65	46	0.88 (0.63-1.19)
	Thyroid	55,572 (37.2)	47.4	58.9	1022 (32.5)	47	1,053	0.97 (0.91-1.03)
	Hodgkin	82 (0.1)	38.4	47.5	2 (0.1)	61	1	1.37 (0.17-4.94)
	Non-Hodgkin	1991 (1.3)	53.1	62.1	57 (1.8)	76	53	1.07 (0.81-1.39)
	Multiple myeloma	457 (0.3)	62.1	67.3	3 (0.1)	29	10	0.30 (0.06-0.87)
	Leukemia	1130 (0.8)	49.0	58.5	17 (0.5)	52	21	0.80 (0.47-1.28)
	Total	149,352 (100)	52.7	63.7	3141 (100)	54	3,542	0.89 (0.86-0.92)

^a^SIR: standardized incidence ratio.

Among FPC types, the risk of developing SPCs was significantly higher after laryngeal cancer in men (SIR 1.28, 95% CI 1.07-1.51; incidence: 248 per 10 000 person-years). For the 3 most common FPC types in 5-year cancer survivors, the overall SIRs in men were significantly lower for stomach, colorectal, and prostate cancers (Table 3). In women, the SIR was significantly lower for stomach cancer (SIR 0.77, 95% CI 0.69-0.86; incidence, 60 per 10 000 person-years) but not significantly lower for breast cancer and thyroid cancer.

Tables S3 and S4 in Multimedia Appendix 1 show the subgroup analyses by age (<40 and ≥40 years) for the risks of developing SPCs by FPC types in men and women. The risk of developing any type of SPC after kidney cancer (SIR 3.02, 95% CI 1.21-6.21) and thyroid cancer (SIR 1.37, 95% CI 1.18-1.58) was significantly higher in young (<40 years) men and women, respectively, than in the general population.

**Figure 2:**
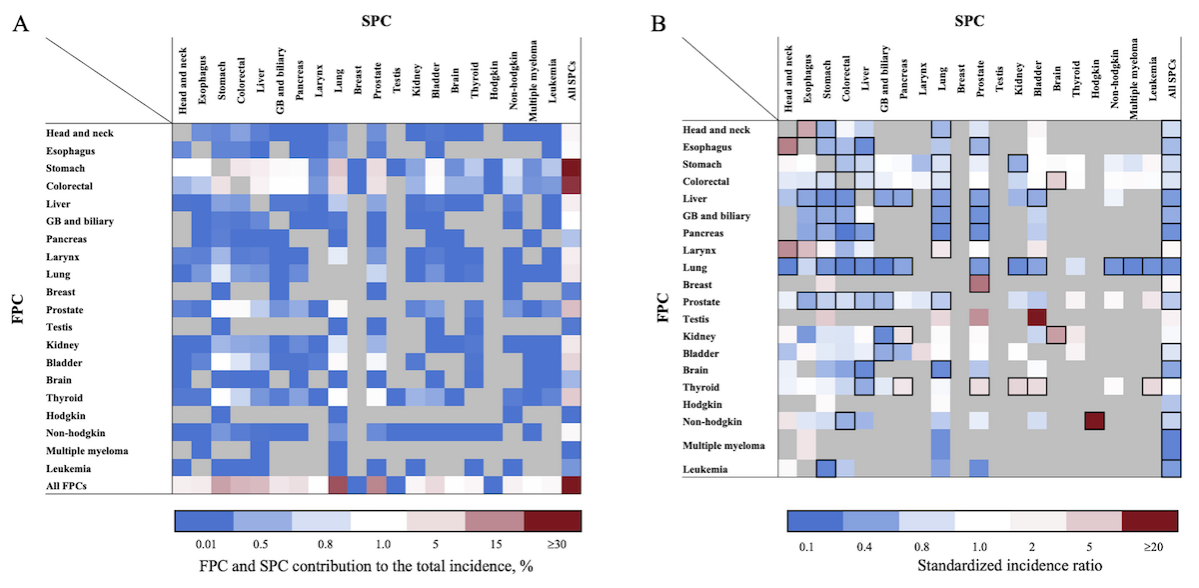
Risk of developing subsequent primary cancers (SPCs) among 5-year male survivors (A) calculated by dividing the observed number of SPCs of each cell by the total number of observed SPCs and the (B) standardized incidence ratios of SPCs by each first primary cancer (FPC). Statistically significant associations between the FPC and SPC are shown by bold boxes, gray cells indicate associations not tested due to a small number of observed SPCs, and blue and red colors represents lower- and higher-than-expected values, respectively, based on 195 eligible statistical tests (observed number of SPCs ≥5 or statistically significant association). GB: gallbladder.

**Figure 3:**
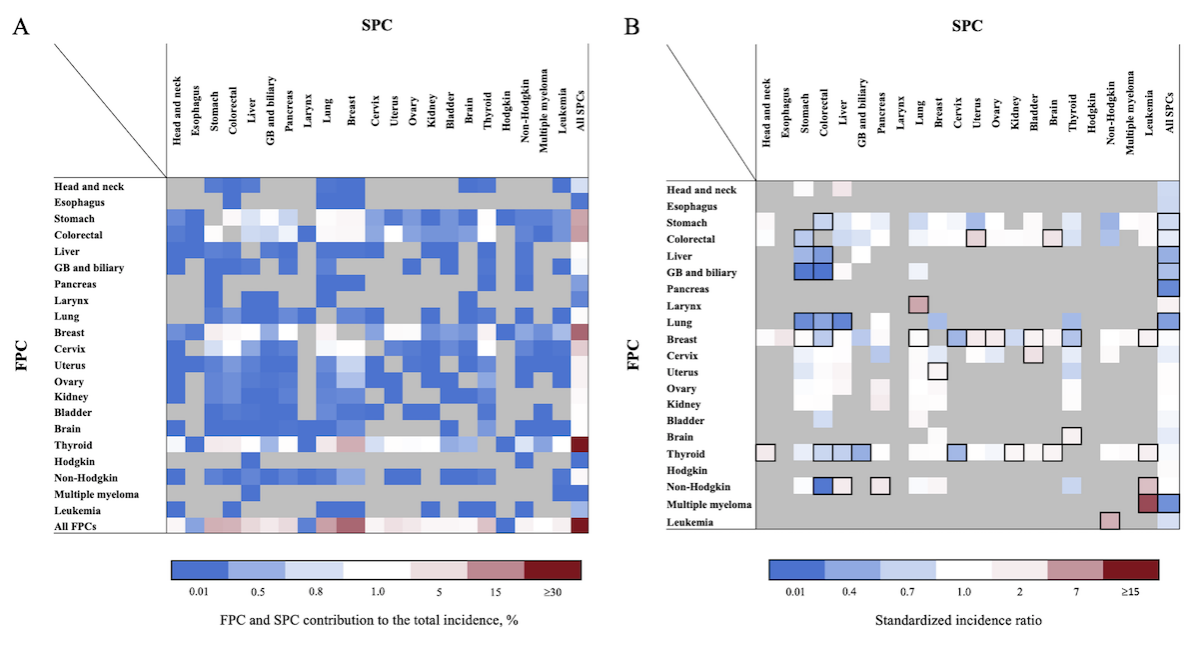
Risk of developing subsequent primary cancers (SPCs) among 5-year female survivors (A) calculated by dividing the observed number of SPCs of each cell by the total number of observed SPCs and the (B) standardized incidence ratios of SPCs by each first primary cancer (FPC). Statistically significant associations between the FPC and SPC are shown by bold boxes, gray cells indicate associations not tested due to a small number of observed SPCs, and blue and red colors represents lower- and higher-than-expected values, respectively, based on 147 eligible statistical tests (observed number of SPCs ≥5 or statistically significant association). GB: gallbladder.

**Figure 4:**
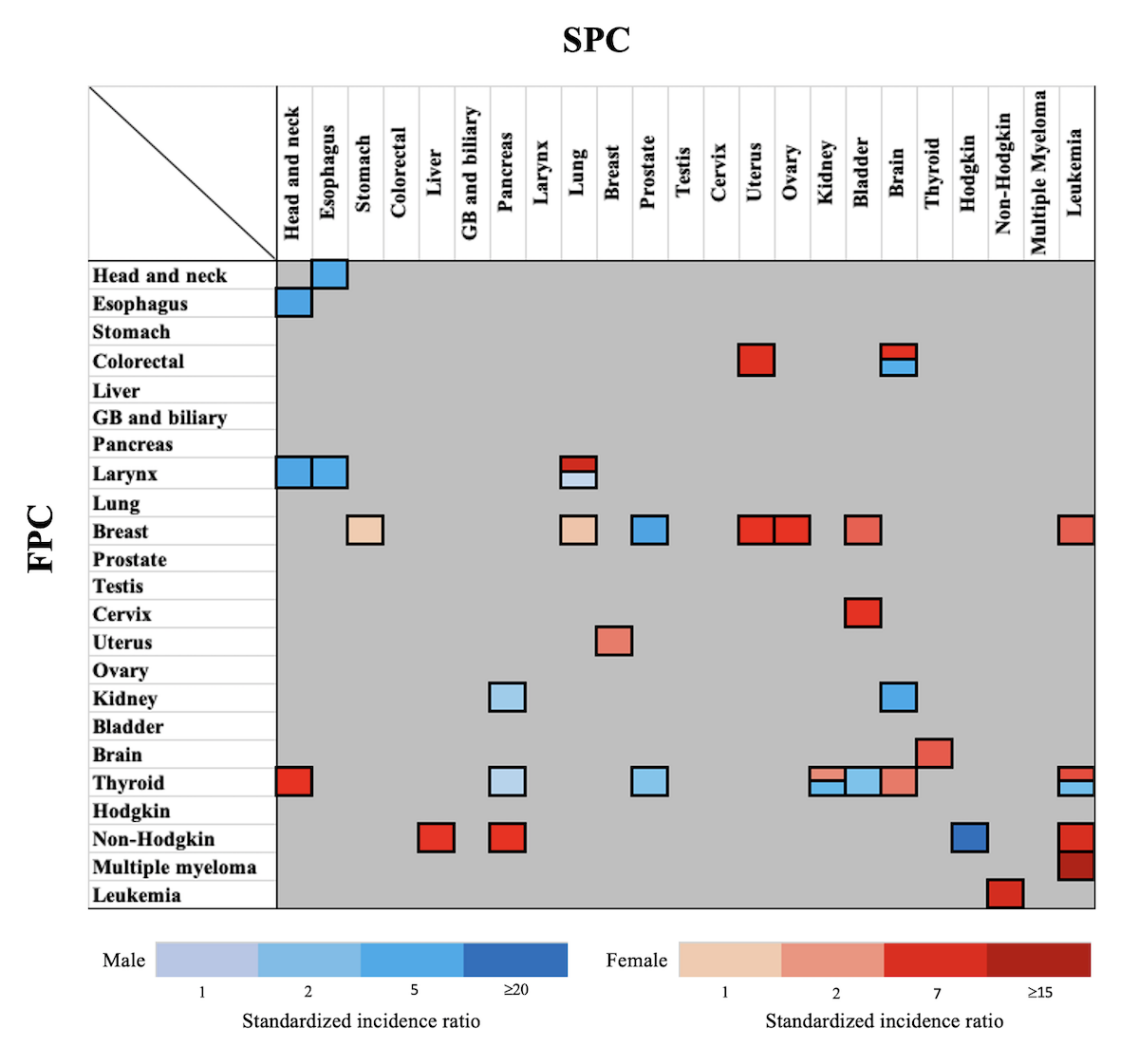
Standardized incidence ratios (SIR; statistically significant associations with an SIR >1) showing the high risk of developing subsequent primary cancers among male and female 5-year survivors for each first primary cancer. FPC: first primary cancer; GB: gallbladder; SPC: subsequent primary cancer.

Figures 2 and 3 display the percentage contributions of different FPC and SPC combinations to the total incidence of SPCs, as well as the SIRs for men and women. Among all survivors, the 5 SPCs that contributed the most to the total incidence of SPCs were lung (21.6%), prostate (15.2%), stomach (12%), colorectal (9.5%), and liver (8.9%) cancers in men and breast (18.9%), lung (12.2%), stomach (10.4%), colorectal (10%), and thyroid (8.2%) cancers in women. These findings are represented in the row labeled “All FPCs” in Figure 2A and Figure 3A. In men, the 5 most significant SIRs for SPCs were observed among survivors of non-Hodgkin lymphoma for Hodgkin lymphoma (SIR 20.6), survivors of breast cancer for prostate cancer (SIR 6.07), survivors of esophageal cancer for head and neck cancer (SIR 5.62), laryngeal cancer survivors for head and neck cancer (SIR 5.19), and survivors of head and neck cancer survivors for esophageal cancer (SIR 4.27). These SIRs are shown in Figure 2B and Multimedia Appendix 2. For women, the 5 most significant SIRs for SPCs were observed among survivors of multiple myeloma for leukemia (SIR 15.11), survivors of laryngeal cancer for lung cancer (SIR 7.83), survivors of leukemia for non-Hodgkin lymphoma (SIR 7.11), survivors of non-Hodgkin lymphoma for leukemia (SIR 5.95), and survivors of colorectal cancer for uterus cancer (SIR 4.25). These findings are shown in Figure 3B and Multimedia Appendix 2. Figure 4 summarizes the heightened risk of SPCs across various FPC and SPC combinations in both men and women. Notably, the risks of brain cancer in colorectal cancer survivors, lung cancer in laryngeal cancer survivors, and both kidney cancer and leukemia in thyroid cancer survivors were significantly higher in both sexes.

The subgroup analysis to assess environmental factors for developing SPCs showed that the risks of developing each factor-related SPC were higher in young (<40 years old) patients with FPCs than older patients in general (Table S5 in Multimedia Appendix 1). Among middle-aged and older (≥40 years) survivors, some types of FPCs were associated with a higher risk of developing SPCs compared with the general population; for example, male survivors of laryngeal cancer had a higher risk of smoking-related SPCs (SIR 1.46, 95% CI 1.20-1.77), and survivors of uterus cancer had a higher risk of obesity-related SPCs (SIR 1.33, 95% CI 1.02-1.71). The results of additional subgroup analyses to assess the contribution of each FPC and SPC to the SIRs by age (<50 years and ≥50 years) in men and women are displayed in Figures S1 and S2 in Multimedia Appendix 3. Some considerable associations between the type of FPC and SPC that could be related to hereditary cancer syndromes such as hereditary breast and ovarian cancer, Lynch syndrome, and Von-Hippel-Lindau (VHL) syndrome were observed in patients with an FPC before 50 years of age (eg, uterus cancer in female breast cancer survivors, uterus cancer in female colorectal cancer survivors, pancreas cancer in male kidney cancer survivors; see Figures S1A and S2A in Multimedia Appendix 3) .

## Discussion

### Principal Findings

The results indicate that the risk of developing SPCs among adult-onset 5-year cancer survivors in South Korea is generally lower than in the general population. However, young (<40 years old) cancer survivors exhibited an increased risk of developing SPCs, and the risk of SPCs also varied according to the patient’s sex and the type of FPC. Survivors from smoking-related cancers have a significant risk of developing subsequent smoking-related cancers. These findings highlight the necessity for tailored approaches to cancer screening, prevention, and management strategies for cancer survivors.

Environmental factors such as smoking, alcohol use, exposure to ultraviolet light, and viral infections are well-known risk factors for the development of cancer [17]. In addition, an estimated 5% to 10% percent of cancers have a heritable component that is caused by genetic susceptibility [18]. Additionally, Tomasetti and Vogelstein [9,19] have suggested that “bad luck,” random mutations occurring during stem cell division, contributes to two-thirds of the adult cancer incidence; based on the results of their statistical model, there is a high correlation between the number of stem cell divisions of a given tissue and the lifetime risk of cancer in that tissue. The epidemiological results indicate a 26% lower risk of developing SPCs (SIR 0.74, 95% CI 0.72-0.76) in the overall sample of Korean cancer survivors when compared with the risk of developing cancer in the general population. This can be explained by the fact that cancer-causing mutations are stochastic in nature; therefore, the probability of “very bad luck,” the development of a SPC after an FPC in cancer survivors, is lower than “bad luck” in the general Korean population.

Although the overall risk of developing SPCs in cancer survivors is low, a high risk of developing well-known smoking-related cancers [20,21] was observed among cancer survivors with smoking-related cancers, including laryngeal, head and neck, and esophageal cancers in men and laryngeal and lung cancers in women. This result highlights the importance of environmental factors in the occurrence of SPCs, and primary prevention should be a priority for controlling both FPCs and SPCs, as a substantial cancer burden could be prevented through lifestyle modifications [22]. Smoking is one of the strongest carcinogens, causing various types of cancer, and quitting smoking has been shown to reduce the risk of cancer and improve survival even after a cancer diagnosis [23,24]. Therefore, it is crucial for cancer survivors to quit smoking to improve their survival and lower their risk of subsequent cancers. However, smoking cessation interventions for cancer survivors have not been successful in the past [25,26]. Clinicians need to communicate with and educate cancer survivors about the high risk of secondary cancer and guide them on smoking cessation. Collaboration between oncologists and primary physicians is essential to provide high-quality comprehensive smoking cessation interventions, as smoking cessation services are mainly provided by primary physicians [27].

The findings of this study indicate that young cancer survivors are at a higher risk of developing SPCs, implying that factors beyond “bad luck,” such as hereditary predispositions, may play a significant role in the development of SPCs in young adults. There were significant associations between specific types of SPCs and FPCs that may be linked to hereditary cancer syndrome. In men, young kidney cancer survivors were at a high risk of developing SPCs, with pancreas and brain cancers being the most common types. VHL syndrome, caused by germline mutations of the VHL gene, is characterized by tumors of the central nervous system, kidney, retina, and pancreas. Only a small number of studies have evaluated VHL syndrome in Korea; the incidence of VHL disease in patients with renal cell carcinoma was reported at 0.7%, and it was male-dominant (70%) with a median age of onset of 33 years (14-59 years) [28,29]. In women, the SIRs were significantly high among breast cancer survivors for ovarian cancer and colorectal cancer survivors for uterus cancers, possibly due to hereditary breast and ovarian cancer and Lynch syndrome harboring a germline mutation in *BRCA1/2* and mismatch repair–related genes, respectively. Identifying hereditary cancer syndrome is crucial for cancer prevention and treatment, as both the individual and one-half of first-degree family members are at high risk of cancer [30]. Hereditary cancer syndromes may represent a broader clinical spectrum than previously understood [31,32]. Further genetic studies targeting young cancer survivors with a high risk of SPCs are worth conducting.

The most common types of FPCs were mainly observed as SPCs in cancer survivors, with lung cancer and prostate cancer in men being the most dominant SPCs. This suggests that cancer screening programs for cancer survivors need to focus more on these cancer types. For example, low-dose computed tomography over chest x-ray for lung cancer screening [33] in both sexes and prostate ultrasonography in addition to prostate-specific antigen tests for male cancer survivors may be worthy of consideration. Furthermore, the risk of SPCs varied by the types of FPC and sex, with young cancer survivors being more vulnerable to developing SPCs. These findings highlight the need for tailored cancer screening programs for this population. Based on these results, there is a need to develop cancer type–, age-, and sex-specific national screening programs for cancer survivors. Further studies are necessary to assess the benefits of new evidence-based tailored screening programs for this population.

An interesting association was observed in male breast cancer survivors, as they had a higher risk of developing prostate cancer as an SPC. The positive association has been reported in male patients [34,35], and 2 hypotheses have been proposed to explain it [36]. The first hypothesis is that 4% to 40% of male breast cancer patients harbor a *BRCA2* germline mutation [37,38]. Since *BRCA1/2* mutations are associated with both breast and prostate cancers, the increased risk of prostate cancer in male breast cancer survivors could be due to their germline susceptibility [39,40]. The second hypothesis is that most male breast cancers are estrogen receptor–positive, which makes them good candidates for hormone treatment with aromatase inhibitors [35,41]. The suppression of estrogen could cause an imbalance of estrogen and testosterone, which hypothetically increases the risk of prostate cancer [42]. This association highlights the need for germline testing and careful prostate cancer screening in male breast cancer survivors.

### Limitations

Although this study provides novel findings on the risk of SPCs in Korean cancer survivors, several limitations need to be acknowledged. Factors such as latency, family cancer history, clinicopathologic and sociodemographic factors, and treatments for FPCs, including chemoradiation therapy, could also influence the risk of SPCs [43]. The results showed strong positive associations among non-Hodgkin lymphoma, Hodgkin lymphoma, and leukemia, which may be due to the effect of chemotherapy [44]. However, due to the unavailability of relevant data, this study did not examine their effects. Additionally, the lack of information on other known risk factors such as smoking, alcohol consumption, diet, physical activity level, excess body weight, environmental carcinogens, and cancer-associated infection status such as human papilloma virus infection, is a limitation. The incidence of SPCs at the same organ as the FPC could be underestimated as they were excluded to minimize bias due to misidentification of FPC recurrence. Furthermore, the incidence of SPCs could be underestimated in cancer survivors with FPC types associated with high mortality, such as pancreatic and lung cancers, as deceased patients within 5 years after the FPC diagnosis were not included. Heightened medical surveillance following the FPC diagnosis may have increased the chance of detecting SPCs, particularly in cases where the sites of the FPC and SPC were anatomically close (eg, thyroid and head and neck or breast and lung cancers). However, to minimize this bias, SPCs occurring within 5 years of the FPC diagnosis were excluded, as ICBP covers the medical cost for only 5 years. Some of the significant associations could be biased by multiple testing, and the statistical power was also limited, especially for the subgroup analysis in young patients with FPCs due to the small sample size. Finally, some minor malignancies that could have been related to genetic susceptibility, such as pheochromocytoma and paraganglioma syndrome [45], were excluded from the analysis as they were classified as other cancers (other C00-96). The American Cancer Society defines cancer survivors as “anyone who has ever been diagnosed with cancer no matter where they are in the course of their disease” [46], whereas we focused on 5-year cancer survivors in this study.

### Comparison With Prior Work

The results of this study differ significantly from those of a previous study that used SEER data [4]. In that study, the risks of SPCs in survivors of adult-onset cancers were higher in several types of primary cancer compared with the general population in the United States. Several factors could explain this difference. First, the same group conducted another study that focused on adolescent and young adult (AYA; aged 15-39 years) cancer survivors using SEER data [47], and the risk of SPCs was generally higher in AYA than in adult cancer survivors (Table S6 in Multimedia Appendix 1). Second, although the SEER study covered 9% to 13% of the US population [4], HIRA covers nearly the entire Korean population. Third, only 8.1% of the population in the SEER study Asian or Pacific Islander descent, while nearly all Koreans are Asian. Fourth, the incidences of cancer and frequent cancer types differ between Korea and the United States (Table S7 in Multimedia Appendix 1) [48]. Fifth, the mean follow-up durations were 7.3 years in the SEER study and 3.8 years in our study, and the shorter follow-up duration in this study might have led to an underestimation of the risk of developing SPCs. Last, cancer screening rates vary by cancer type and nation [49,50].

### Conclusion

The varying risk of SPCs by age, sex, and the type of FPC in cancer survivors suggests the necessity for ongoing efforts to develop tailored prevention and screening programs for cancer survivors. Lifestyle modifications, including smoking cessation, are essential to reduce the risk of SPCs in cancer survivors. In addition, genetic testing, along with proactive cancer screening and prevention strategies, should be implemented for young cancer survivors because of their elevated risk of developing SPCs.

## Appendix

Multimedia Appendix 1 Supplementary tables.

Multimedia Appendix 2 The details of the risk of developing subsequent primary cancers (SPCs) among 5-year male survivors by types of first primary cancer (FPC).

Multimedia Appendix 3 Supplementary figures.

## Abbreviations

AYA: adolescent and young adult
FPC: first primary cancer
HIRA: Health Insurance Review and Assessment
ICBP: Korean Individual Co-payment Beneficiaries Program
*ICD*: *International Classification of Diseases*
SEER: Surveillance, Epidemiology, and End Results Program
SIR: standardized incidence ratio
SPC: subsequent primary cancer
VHL: Von-Hippel-Lindau

## Data Availability

Data sharing is not applicable to this article, as the Health Insurance Review and Assessment (HIRA) data are managed by the Korean government. Access to the data is restricted to authorized individuals after review of the study protocol and is only permissible from designated computers.
