# Supplementary material for: Risk of Subsequent Primary Cancers Among Adult-Onset 5-Year Cancer Survivors in South Korea: Retrospective Cohort Study
Source: JMIR Public Health Surveill. 2024 May 8;10:e48380. doi: 10.2196/48380 (PMC11112468; doi:10.2196/48380)
Supplement: Multimedia Appendix 1 [file publichealth_v10i1e48380_app1.docx]

**Table S1. The numbers of patients with first primary cancer and 5-year survivors, categorized by the type of the first primary cancer.**

| ICD | Type of First Primary cancer | Patients, No. (%) | Survivors, No. (%) | % of 5-year survivors of first primary cancer |
| --- | --- | --- | --- | --- |
| C00-14 | Head & Neck | 5,534 (1.5) | 3,491 (1.3) | 63.1 |
| C15 | Esophagus | 3,702 (1.0) | 1,596 (0.6) | 43.1 |
| C16 | Stomach | 61,775 (16.6) | 45,347 (17.0) | 73.4 |
| C18-20 | Colo-rectum | 49,614 (13.4) | 36,618 (13.8) | 73.8 |
| C22 | Liver | 28,858 (7.8) | 13,178 (5.0) | 45.7 |
| C23-24 | GB & Biliary | 7,332 (2.0) | 2,683 (1.0) | 36.6 |
| C25 | Pancreas | 7,392 (2.0) | 2,628 (1.0) | 35.6 |
| C32 | Larynx | 2,468 (0.7) | 1,540 (0.6) | 62.4 |
| C33-34 | Lung | 32,182 (8.7) | 14,559 (5.5) | 45.2 |
| C50 | Breast | 34,706 (9.4) | 29,977 (11.3) | 86.4 |
| C53 | Cervix | 11,144 (3.0) | 8,901 (3.3) | 79.9 |
| C54 | Uterus | 3,642 (1.0) | 2,529 (1.0) | 69.4 |
| C56 | Ovary | 4,680 (1.3) | 3,089 (1.2) | 66.0 |
| C61 | Prostate | 13,539 (3.7) | 10,628 (4.0) | 78.5 |
| C62 | Testis | 493 (0.1) | 408 (0.2) | 82.8 |
| C64 | Kidney | 7,065 (1.9) | 5,221 (2.0) | 73.9 |
| C67 | Bladder | 8,592 (2.3) | 6,179 (2.3) | 71.9 |
| C70-72 | Brain | 5,254 (1.4) | 3,500 (1.3) | 66.6 |
| C73 | Thyroid | 69,211 (18.7) | 66,087 (24.8) | 95.5 |
| C81 | Hodgkin lymphoma | 455 (0.1) | 246 (0.1) | 54.1 |
| C82-86, C96 | Non-Hodgkin lymphoma | 7,001 (1.9) | 4,349 (1.6) | 62.1 |
| C90 | Multiple myeloma | 1,942 (0.5) | 948 (0.4) | 48.8 |
| C91-95 | Leukemia | 4,600 (1.2) | 2,539 (1.0) | 55.2 |
|  | Total | 371,181 (100.0) | 266,241 (100.0) | 71.7 |

**Table S2.** **The risk of developing any type of subsequent primary cancers by age among 5-year cancer survivors with their first diagnosis between 2009 and 2010 in South Korea.** (bold font indicates statistical significancy)

| Age at FPC diagnosis, year | Survivors, No. (%) | observed SPC, No. (%) | expected SPC | SIR (95% CI) |
| --- | --- | --- | --- | --- |
| 18-19 | 670 (0.3) | 4 (0.1) | 1 | 3.38 (0.92 to 8.66) |
| 20-24 | 1,788 (0.7) | 14 (0.2) | 5 | **2.55 (1.39 to 4.28)** |
| 25-29 | 5,036 (1.9) | 45 (0.6) | 27 | **1.69 (1.23 to 2.26)** |
| 30-34 | 9,373 (3.5) | 104 (1.4) | 77 | **1.34 (1.1 to 1.63)** |
| 35-39 | 16,304 (6.1) | 214 (2.9) | 186 | **1.15 (1.0 to 1.31)** |
| 40-44 | 23,132 (8.7) | 336 (4.6) | 338 | 0.99 (0.89 to 1.11) |
| 45-49 | 31,557 (11.9) | 540 (7.3) | 590 | 0.92 (0.84 to 1) |
| 50-54 | 36,652 (13.8) | 846 (11.5) | 881 | 0.96 (0.9 to 1.03) |
| 55-59 | 30,936 (11.6) | 860 (11.7) | 1,029 | **0.84 (0.78 to 0.89)** |
| 60-64 | 30,544 (11.5) | 1,162 (15.8) | 1,414 | **0.82 (0.78 to 0.87)** |
| 65-69 | 32,129 (12.1) | 1,392 (18.9) | 1,934 | **0.72 (0.68 to 0.76)** |
| 70-74 | 27,966 (10.5) | 1,156 (15.7) | 1,962 | **0.59 (0.56 to 0.62)** |
| 75-79 | 17,688 (6.6) | 607 (8.3) | 1,292 | **0.47 (0.43 to 0.51)** |
| ≥ 80 | 2,466 (0.9) | 68 (0.9) | 175 | **0.39 (0.3 to 0.49)** |
| Total | 266,241(100.0) | 7,348 (100.0) | 9,911 | **0.74 (0.72 to 0.76)** |

SPCs; subsequent primary cancers, SIR; standardized incidence ratio

**Table S3. The subgroup analysis by age at first primary cancer for the risk of developing any type of subsequent primary cancer among male 5-year cancer survivors, categorized by the type of the first primary cancer.**

|  | Age at FPC diagnosis <40 | | | | Age at FPC diagnosis ≥40 | | | |
| --- | --- | --- | --- | --- | --- | --- | --- | --- |
| **FPC** | Observed SPCs, No. | Incidence per 10,000 person-years | Expected SPCs, No. | SIR (95% CI) | Observed SPCs, No. | Incidence per 10,000 person-years | Expected SPCs, No. | SIR (95% CI) |
| Head & Neck | 4 | 41 | 1.4 | 2.84 (0.77 to 7.26) | 91 | 117 | 125.1 | **0.73 (0.59 to 0.89)** |
| Esophagus | 0 | 0 | 0.0 | 0 | 63 | 111 | 119.8 | **0.53 (0.40 to 0.67)** |
| Stomach | 8 | 19 | 6.5 | 1.23 (0.53 to 2.42) | 1,278 | 111 | 1,743.8 | **0.73 (0.69 to 0.77)** |
| Colo-rectum | 10 | 31 | 5.1 | 1.98 (0.95 to 3.63) | 1,061 | 130 | 1,317.2 | **0.81 (0.76 to 0.86)** |
| Liver | 0 | 0 | 2.0 | 0 | 129 | 44 | 435.9 | **0.30 (0.25 to 0.35)** |
| GB & Biliary | 1 | 148 | 0.1 | 7.79 (0.20 to 43.41) | 43 | 85 | 105.3 | **0.41 (0.30 to 0.55)** |
| Pancreas | 1 | 74 | 0.2 | 4.63 (0.12 to 25.81) | 25 | 44 | 115.4 | **0.22 (0.14 to 0.32)** |
| Larynx | 1 | 136 | 0.1 | 6.83 (0.17 to 38.06) | 131 | 249 | 103.2 | **1.27 (1.06 to 1.51)** |
| Lung | 0 | 0 | 0.8 | 0 | 158 | 40 | 710.7 | **0.22 (0.19 to 0.26)** |
| Breast | 0 | 0 | 0.0 | 0 | 8 | 205 | 5.9 | 1.35 (0.58 to 2.66) |
| Prostate | 0 | 0 | 0.1 | 0 | 362 | 133 | 565.7 | **0.64 (0.58 to 0.71)** |
| Testis | 2 | 16 | 1.5 | 1.36 (0.17 to 4.92) | 4 | 112 | 2.5 | 1.58 (0.43 to 4.05) |
| Kidney | 7 | 49 | 2.3 | **3.02 (1.21 to 6.21)** | 149 | 137 | 161.0 | 0.93 (0.78 to 1.09) |
| Bladder | 1 | 17 | 1.0 | 0.99 (0.03 to 5.49) | 251 | 160 | 305.8 | **0.82 (0.72 to 0.93)** |
| Brain | 1 | 5 | 2.2 | 0.46 (0.01 to 2.54) | 22 | 57 | 57.8 | **0.38 (0.24 to 0.58)** |
| Thyroid | 18 | 15 | 15.7 | 1.15 (0.68 to 1.81) | 282 | 100 | 271.3 | 1.04 (0.92 to 1.17) |
| Hodgkin | 0 | 0 | 0.3 | 0 | 3 | 87 | 4.7 | 0.64 (0.13 to 1.87) |
| Non-Hodgkin | 6 | 33 | 2.3 | 2.60 (0.95 to 5.65) | 70 | 102 | 107.3 | **0.65 (0.51 to 0.82)** |
| Multiple myeloma | 0 | 0 | 0.1 | 0 | 3 | 28 | 23.3 | **0.13 (0.03 to 0.38)** |
| Leukemia | 2 | 14 | 1.5 | 1.32 (0.16 to 4.78) | 12 | 46 | 44.4 | **0.27 (0.14 to 0.47)** |
| Total | 62 | 20 | 43.2 | **1.43 (1.10 to 1.84)** | 4,145 | 106 | 6,326.2 | **0.66 (0.64 to 0.68)** |

FPC, first primary cancers, SPCs; subsequent primary cancers, SIR; standardized incidence ratio

**Table S4. The subgroup analysis by age at first primary cancer for the risk of developing any type of subsequent primary cancer among female 5-year cancer survivors, categorized by the type of first primary cancer.**

| Women | Age at FPC diagnosis <40 | | | | Age at FPC diagnosis ≥ 40 | | | |
| --- | --- | --- | --- | --- | --- | --- | --- | --- |
| **FPC** | Observed SPCs, No. | Incidence per 10,000 person-years | Expected SPCs, No. | SIR (95% CI) | Observed SPCs, No. | Incidence per 10,000 person-years | Expected SPCs, No. | SIR (95% CI) |
| Head & Neck | 6 | 71 | 2.6 | 2.34 (0.86 to 5.08) | 19 | 53 | 30.2 | **0.63 (0.38 to 0.98)** |
| Esophagus | 0 | 0 | 0.0 | 0 | 4 | 76 | 5.3 | 0.76 (0.21 to 1.94) |
| Stomach | 13 | 32 | 14.6 | 0.89 (0.47 to 1.52) | 353 | 62 | 459.9 | **0.77 (0.69 to 0.85)** |
| Colo-rectum | 12 | 57 | 7.6 | 1.58 (0.82 to 2.76) | 382 | 69 | 440.1 | **0.87 (0.78 to 0.96)** |
| Liver | 0 | 0 | 1.3 | 0 | 41 | 44 | 82.5 | **0.50 (0.36 to 0.67)** |
| GB & Biliary | 2 | 215 | 0.3 | 5.77 (0.70 to 20.83) | 28 | 54 | 50.2 | **0.56 (0.37 to 0.81)** |
| Pancreas | 1 | 36 | 0.8 | 1.19 (0.03 to 6.60) | 11 | 23 | 46.9 | **0.24 (0.12 to 0.42)** |
| Larynx | 0 | 0 | 0.1 | 0 | 7 | 205 | 3.3 | 2.11 (0.85 to 4.35) |
| Lung | 1 | 21 | 1.7 | 0.60 (0.02 to 3.32) | 48 | 34 | 126.5 | **0.38 (0.28 to 0.50)** |
| Breast | 34 | 23 | 39.1 | 0.87 (0.60 to 1.22) | 565 | 57 | 568.8 | 0.99 (0.91 to 1.08) |
| Cervix | 26 | 38 | 23.6 | 1.10 (0.72 to 1.61) | 196 | 66 | 225.0 | 0.87 (0.75 to 1.00) |
| Uterus | 5 | 41 | 4.3 | 1.17 (0.38 to 2.72) | 75 | 84 | 63.3 | 1.19 (0.93 to 1.49) |
| Ovary | 15 | 46 | 9.7 | 1.55 (0.87 to 2.56) | 65 | 84 | 58.3 | 1.11 (0.86 to 1.42) |
| Kidney | 1 | 17 | 2.1 | 0.48 (0.01 to 2.69) | 53 | 90 | 50.3 | 1.05 (0.79 to 1.38) |
| Bladder | 0 | 0 | 0.5 | 0 | 37 | 97 | 38.4 | 0.97 (0.68 to 1.38) |
| Brain | 6 | 48 | 3.3 | 1.81 (0.67 to 3.94) | 34 | 69 | 42.4 | 0.80 (0.56 to 1.12) |
| Thyroid | 184 | 32 | 134.2 | **1.37 (1.18 to 1.58)** | 838 | 53 | 919.1 | **0.91 (0.85 to 0.98)** |
| Hodgkin | 1 | 49 | 0.5 | 1.90 (0.05 to 10.56) | 1 | 81 | 0.9 | 1.07 (0.03 to 5.95) |
| Non-Hodgkin | 7 | 46 | 4.4 | 1.58 (0.64 to 3.25) | 50 | 84 | 48.7 | 1.03 (0.76 to 1.35) |
| Multiple myeloma | 0 | 0 | 0.1 | 0 | 3 | 30 | 10.0 | **0.30 (0.06 to 0.88)** |
| Leukemia | 5 | 48 | 2.7 | 1.82 (0.59 to 4.26) | 12 | 54 | 18.5 | 0.65 (0.34 to 1.13) |
| Total | 319 | 33 | 253.7 | **1.26 (1.12 to 1.40)** | 2,822 | 59 | 3,288.5 | **0.86 (0.83 to 0.89)** |

FPC, first primary cancers, SPCs; subsequent primary cancers, SIR; standardized incidence ratio

**Table S5. Risk of developing any smoking-, obesity-, alcohol-, or infection-related subsequent primary cancer among survivors of prior smoking-, obesity-, alcohol-, or infection-related cancers.**

|  | | Age at FPC diagnosis < 40 | | | | Age at FPC diagnosis ≥ 40 | | | |
| --- | --- | --- | --- | --- | --- | --- | --- | --- | --- |
|  |  | **Incidence per 10,000 person-years** | | **SIRs (95%CI)** | | **Incidence per 10,000 person-years** | | **SIRs (95%CI)** | |
|  |  | **Men** | **Women** | **Men** | **Women** | **Men** | **Women** | **Men** | **Women** |
| Smoking-related cancers (n=12) | Head & Neck | 20 | 36 | 2.45 (0.30 to 8.86) | **5.38 (1.11 to 15.74)** | 91 | 36 | **0.78 (0.61 to 0.99)** | 0.80 (0.42 to 1.36) |
|  | Esophagus | 0 | 0 | 0 | 0 | 99 | 38 | **0.65 (0.49 to 0.85)** | 0.63 (0.08 to 2.28) |
|  | Stomach | 14 | 7 | 1.66 (0.61 to 3.61) | 1.16 (0.24 to 3.39) | 78 | 34 | **0.76 (0.71 to 0.81)** | **0.82 (0.71 to 0.94)** |
|  | Colon and Rectum | 18 | 14 | 2.09 (0.77 to 4.54) | 2.08 (0.43 to 6.09) | 90 | 36 | **0.82 (0.76 to 0.88)** | 0.9 (0.78 to 1.03) |
|  | Liver | 0 | 0 | 0 | 0 | 31 | 27 | **0.30 (0.24 to 0.37)** | **0.54 (0.35 to 0.80)** |
|  | Pancreas | 74 | 0 | 7.71 (0.2 to 43.0) | 0 | 33 | 13 | **0.23 (0.14 to 0.36)** | **0.22 (0.08 to 0.47)** |
|  | Larynx | 0 | 0 | 0 | 0 | 207 | 146 | **1.46 (1.2 to 1.77)** | 2.55 (0.83 to 5.94) |
|  | Lung | 0 | 0 | 0 | 0 | 28 | 20 | **0.24 (0.2 to 0.29)** | **0.42 (0.28 to 0.6)** |
|  | Cervix | NA | 6 | NA | 0.93 (0.25 to 2.37) | NA | 40 | NA | 1.09 (0.90 to1.30) |
|  | Kidney | 35 | 0 | **3.61 (1.17 to 8.43)** | 0 | 104 | 60 | 0.97 (0.80 to 1.17) | 1.27 (0.89 to 1.77) |
|  | Bladder | 17 | 0 | 1.61 (0.04 to 8.99) | 0 | 128 | 63 | 0.92 (0.80 to 1.06) | 1.04 (0.67 to 1.55) |
|  | Non-lymphocytic leukemia | 24 | 0 | 4.08 (0.49 to 14.75) | 0 | 56 | 47 | **0.46 (0.21 to 0.87)** | 1.07 (0.43 to 2.21) |
| Obesity-related cancers (n=12) | GB & Biliary | 148 | 215 | 10.84 (0.27 to 60.41) | 6.82 (0.83 to 24.63) | 55 | 27 | **0.56 (0.37 to 0.81)** | **0.44 (0.24 to 0.73)** |
|  | Pancreas | 74 | 0 | 6.51 (0.16 to 36.29) | 0 | 24 | 15 | **0.26 (0.14 to 0.43)** | **0.23 (0.09 to 0.48)** |
|  | Stomach | 14 | 27 | 1.39 (0.51 to 3.03) | 0.90 (0.45 to 1.61) | 47 | 45 | **0.72 (0.66 to 0.78)** | **0.88 (0.77 to 0.99)** |
|  | Thyroid | 9 | 25 | 1.09 (0.54 to 1.95) | **1.40 (1.18 to 1.65)** | 49 | 38 | 0.90 (0.76 to 1.06) | 0.94 (0.87 to 1.02) |
|  | Kidney | 28 | 0 | 2.45 (0.67 to 6.26) | 0 | 71 | 72 | 0.91 (0.72 to 1.13) | 1.20 (0.87 to 1.63) |
|  | Esophagus | 0 | 0 | 0 | 0 | 41 | 57 | **0.39 (0.25 to 0.58)** | 0.86 (0.18 to 2.50) |
|  | Colon and Rectum | 22 | 38 | 2.04 (0.82 to 4.20) | 1.26 (0.54 to 2.47) | 58 | 49 | **0.81 (0.74 to 0.89)** | 0.99 (0.88 to 1.11) |
|  | Uterus |  | 41 | NA | 1.39 (0.45 to 3.23) |  | 69 | NA | **1.33 (1.02 to 1.71)** |
|  | Breast | 0 | 17 | 0 | 0.83 (0.54 to 1.22) | 77 | 42 | 0.95 (0.20 to 2.78) | 1.06 (0.96 to 1.16) |
|  | Ovary |  | 40 | NA | 1.61 (0.86 to 2.75) |  | 68 | NA | 1.26 (0.94 to 1.65) |
|  | Multiple myeloma | 0 | 0 | 0 | 0 | 19 | 10 | **0.17 (0.02 to 0.63)** | **0.15 (0.01 to 0.85)** |
|  | Liver | 0 | 0 | 0 | 0 | 19 | 30 | **0.27 (0.20 to 0.35)** | **0.52 (0.34 to 0.75)** |
| Alcohol-related cancers (n=7) | Head & Neck | 10 | 36 | 1.67 (0.04 to 9.3) | 2.82 (0.58 to 8.23) | 62 | 34 | 0.88 (0.65 to 1.17) | 0.88 (0.45 to 1.54) |
|  | Esophagus | 0 | 0 | 0 | 0 | 56 | 57 | **0.65 (0.44 to 0.92)** | 1.27 (0.26 to 3.71) |
|  | stomach | 10 | 12 | 1.67 (0.45 to 4.27) | 0.81 (0.26 to 1.90) | 34 | 27 | **0.69 (0.62 to 0.76)** | 0.88 (0.75 to 1.03) |
|  | Colon and Rectum | 9 | 10 | 1.51 (0.31 to 4.41) | 0.61 (0.07 to 2.21) | 44 | 25 | **0.78 (0.70 to 0.87)** | 0.88 (0.74 to 1.04) |
|  | Liver | 0 | 0 | 0 | 0 | 15 | 18 | **0.25 (0.18 to 0.34)** | **0.49 (0.29 to 0.79)** |
|  | Larynx | 0 | 0 | 0 | 0 | 105 | 58 | 1.25 (0.94 to 1.62) | 1.33 (0.16 to 4.79) |
|  | Breast | 0 | 5 | 0 | 0.91 (0.37 to 1.87) | 51 | 20 | 0.76 (0.09 to 2.73) | 1.02 (0.89 to 1.18) |
| Infection-related cancers (n=6) | Head & Neck | 0 | 36 | 0 | **8.20 (1.69 to 23.95)** | 27 | 20 | **0.60 (0.37 to 0.92)** | 1.03 (0.42 to 2.13) |
|  | Stomach | 7 | 5 | 1.95 (0.40 to 5.70) | 1.50 (0.18 to 5.43) | 18 | 9 | **0.81 (0.71 to 0.93)** | 0.86 (0.64 to 1.12) |
|  | Liver | 0 | 0 | 0 | 0 | 10 | 14 | **0.31 (0.21 to 0.44)** | 0.81 (0.43 to 1.39) |
|  | Cervix |  | 3 | NA | 0.84 (0.10 to 3.05) |  | 15 | NA | 1.04 (0.76 to 1.38) |
|  | Hodkin lymphoma | 0 | 49 | 0 | 12.78 (0.32 to 71.18) | 58 | 81 | 1.41 (0.17 to 5.10) | 4.97 (0.13 to 27.67) |
|  | Non-Hodgkin lymphoma | 11 | 20 | 2.81 (0.34 to 10.14) | **4.90 (1.01 to 14.32)** | 33 | 19 | 0.76 (0.48 to 1.15) | 1.06 (0.53 to 1.89) |

**Table S6. Comparison of the risk of subsequent primary cancer between 5-year survivors of adolescent and young adult (AYA) cancers and adult cancers in the Unites States, using data from the Surveillance, Epidemiology, and End Results Program (SEER) registries.** The table shows that the risk of subsequent primary cancers was generally higher among AYA survivors.

|  | **SIRs (95% CI)** | | |
| --- | --- | --- | --- |
|  | **AYA [47]** | **Male^*^ [4]** | **Female^*^ [4]** |
| **Overall** | 1.25 (1.23 to 1.27) | 1.11 (1.10 to 1.12) | 1.10 (1.09 to 1.11) |
| **Breast (F)** | 1.31 (1.25 to 1.37) | 1.21 (1.08 to 1.35) | 1.06 (1.05 to 1.08) |
| **Melanoma** | 0.87 (0.83 to 0.92) | 0.92 (0.90 to 0.95) | 0.97 (0.94 to 1.01) |
| **Thyroid** | 1 (0.95 to 1.05) | 1.14 (1.08 to 1.20) | 1.10 (1.07 to 1.15) |
| **Testis** | 1.21 (1.14 to 1.27) | 1.05 (0.99 to 1.13) | NA |
| **Cervix Uteri** | 1.05 (0.99 to 1.12) | NA | 1.12 (1.07 to 1.18) |
| **Non-Hodgkin lymphoma** | 1.64 (1.53 to 1.76) | 1.16 (1.14 to 1.20) | 1.21 (1.17 to 1.25) |
| **Hodgkin lymphoma (M)** | 2.24 (2.09 to 2.41) | 1.59 (1.47 to 1.72) | NA |
| **Hodgkin lymphoma (F)** | 3.05 (2.88 to 3.24) | NA | 1.86 (1.71 to 2.02) |
| **Brain and CNS** | 1.11 (0.97 to 1.25) | 1.05 (0.93 to 1.19) | 0.97 (0.84 to 1.12) |
| **Colon and rectum** | 1.36 (1.25 to 1.48) | 0.99 (0.98 to 1.02) | 1.04 (1.02 to 1.07) |
| **Head and Neck** | 1.18 (1.07 to 1.3) | 1.34 (1.30 to 1.39) | 1.39 (1.31 to 1.47) |
| **Ovary** | 1.19 (1.07 to 1.33) | NA | 1.12 (1.07 to 1.19) |
| **Corpus Uteri** | 1.19 (1.07 to 1.33) | NA | 1.03 (1.01 to 1.06) |
| **Soft tissue including heart** | 1.26 (1.11 to 1.43) | 1.10 (1.02 to 1.20) | 1.14 (1.04 to 1.27) |
| **Kidney and renal pelvis** | 1.26 (1.1 to 1.45) | 1.08 (1.04 to 1.12) | 1.16 (1.10 to 1.22) |
| **Urinary bladder** | 0.99 (0.88 to 1.1) | 1.08 (1.06 to 1.11) | 1.23 (1.18 to 1.29) |
| **Bones and joints** | 1.48 (1.25 to 1.74) | 1.02 (0.84 to 1.24) | 0.88 (0.70 to 1.11) |
| **Kaposi sarcoma (M)** | 2.58 (2.18 to 3.03) | 1.54 (1.40 to 1.70) | 1.33 (0.78 to 2.14) |
| **Acute myeloid leukemia** | 1.68 (1.36 to 2.06) |  | 1.18 (0.99 to 1.40) |
| **Lung and bronchus** | 1.06 (0.87 to 1.28) | 1.20 (1.16 to 1.26) | 1.17 (1.12 to 1.23) |
| **Chronic myeloid leukemia** | 1.65 (1.26 to 2.13) | 1.18 (1.03 to 1.34) | 1.04 (0.86 to 1.23) |
| **Vagina and vulva** | 1.09 (0.89 to 1.32) | NA | 1.21 (1.12 to 1.31) |
| **Acute lymphocytic leukemia** | 1.77 (1.33 to 2.3) | 1.31 (0.92 to 1.82) | 1.53 (1.05 to 2.17) |
| **Stomach** | 1.34 (0.98 to 1.79) | 1.13 (1.05 to 1.21) | 1.00 (0.90 to 1.11) |
| **Eye and orbit** | 1.16 (0.85 to 1.54) | 1.13 (0.99 to 1.29) | 1.12 (0.95 to 1.33) |
| **Myeloma** | 1.95 (1.34 to 2.76) | 1.01 (0.93 to 1.10) | 1.03 (0.93 to 1.16) |
| **Small intestine** | 1.67 (1.15 to 2.35) | 1.05 (0.94 to 1.18) | 1.19 (1.04 to 1.35) |
| **Pancreas** | 2.04 (1.35 to 2.94) | 1.38 (1.19 to 1.60) | 1.37 (1.16 to 1.62) |
| **Anus** | 2.14 (1.53 to 2.91) | 1.17 (1.04 to 1.33) | 1.44 (1.30 to 1.60) |

AYA: Adolescent and young adult (ages 15-39 years)

**^*^** Ages 20-84 years

**Table S7. Age-standardized cancer incidence rates from South Korea and United States, presented in units of rates per 100,000 population [48]**.

|  | Men | | | | Women | | | |
| --- | --- | --- | --- | --- | --- | --- | --- | --- |
| Rank | South Korea (2019) | | Unites States (2020) | | South Korea (2019) | | Unites States (2020) | |
|  | All cancers | 296.6 | All cancers | 400.9 | All cancers | 267.4 | All cancers | 333.2 |
| 1 | Stomach | 42.4 | Prostate | 72 | Thyroid | 65.6 | Breast | 90.3 |
| 2 | Lung | 41.7 | Lung | 36.6 | Breast | 60.5 | Lung | 30.4 |
| 3 | Colo-rectum | 37 | Colo-rectum | 28.7 | Colo-rectum | 21.4 | Colo-rectum | 22.9 |
| 4 | Prostate | 34.3 | Melanoma | 19.2 | Stomach | 18.4 | Melanoma | 21.4 |
| 5 | Liver | 25 | Bladder | 18.3 | Lung | 16.9 | Thyroid | 17.4 |
