# Supplementary material for: Risk of Subsequent Primary Cancers Among Adult-Onset 5-Year Cancer Survivors in South Korea: Retrospective Cohort Study
Source: JMIR Public Health Surveill. 2024 May 8;10:e48380. doi: 10.2196/48380 (PMC11112468; doi:10.2196/48380)

**Figure S1.** **Risk of developing subsequent primary cancers among 5-year male survivors, with patients divided into two groups: those with first primary cancer (FPC) before 50 years (A) and those with FPC ≥ 50 years (B).** The figure displays the standardized incidence ratios for subsequent primary cancers (SPCs) for each FPC. Bold boxes indicate statistically significant association between the FPC and SPC. Logarithmic color gradients indicate SIRs, with blue and red colors representing lower and higher than expected values based on 22 and 136 of eligible statistical tests (observed number of SPCs ≥5 or statistically significant association), respectively. Gray cells indicate associations that were not tested due to small number of observed SPCs.


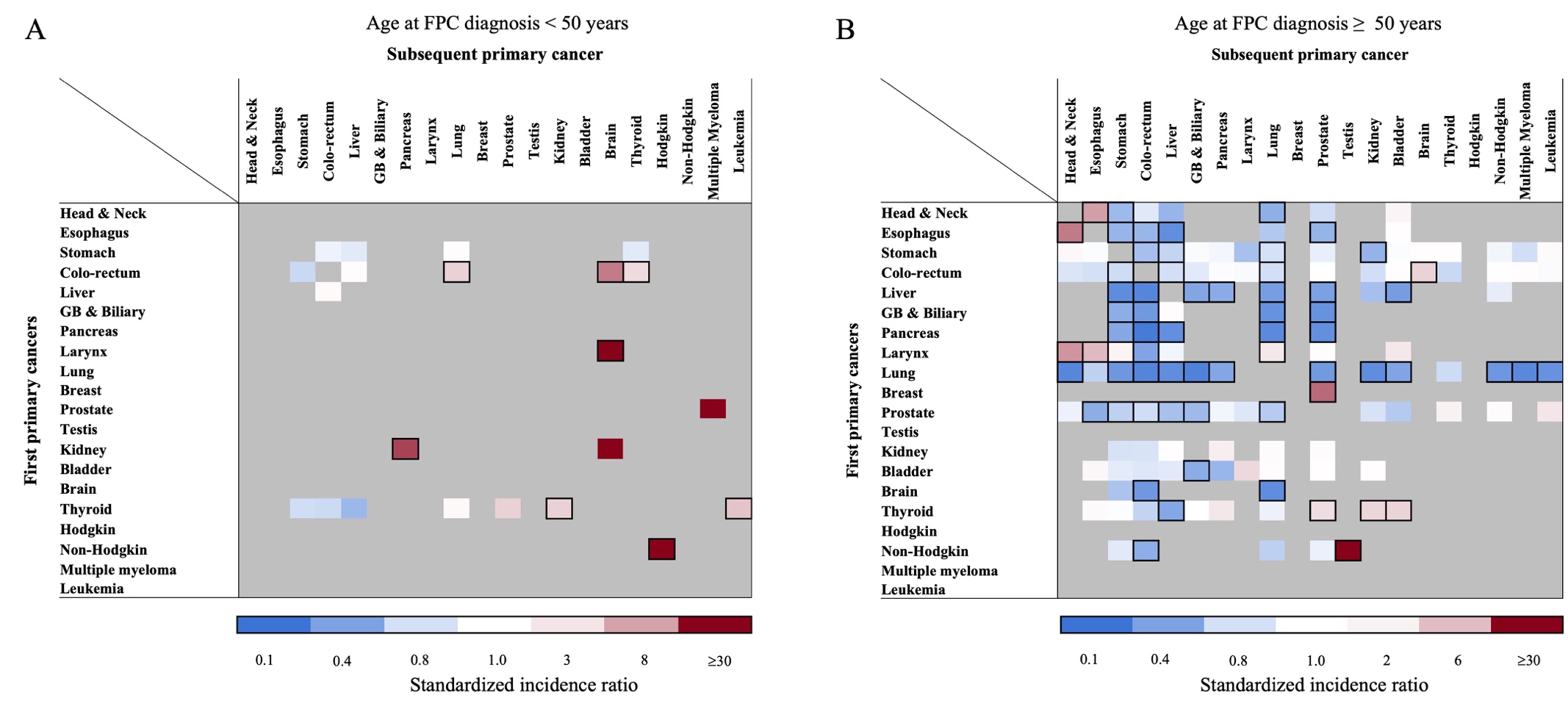


**Figure S2.** **Risk of developing subsequent primary cancers among 5-year female survivors, with patients divided into 2 groups: those with first primary cancer (FPC) before 50 years (A) and those with FPC ≥50 years (B).** The figure displays the standardized incidence ratios for subsequent primary cancers (SPCs) for each FPC. Bold boxes indicate statistically significant association between the FPC and SPC. Logarithmic color gradients indicate SIRs, with blue and red colors representing lower and higher than expected values based on 49 and 115 of eligible statistical tests (observed number of SPCs ≥5 or statistically significant association), respectively. Gray cells indicate associations that were not tested due to small number of observed SPCs.
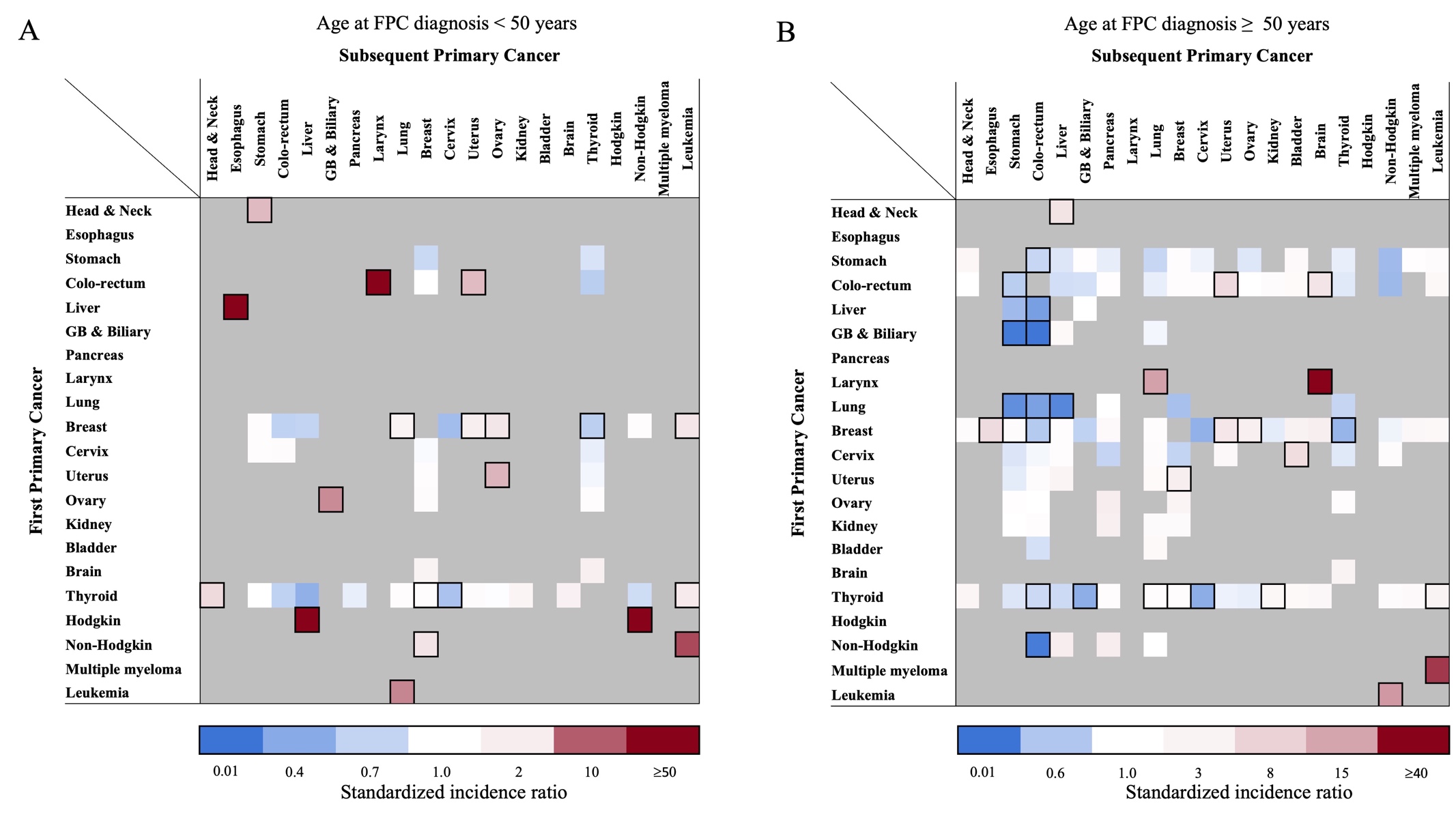

Supplement: Multimedia Appendix 3 [file publichealth_v10i1e48380_app3.docx]
